# Herb–Drug Interaction Potential of Anti-Borreliae Effective Extracts from *Uncaria tomentosa* (Samento) and *Otoba parvifolia* (Banderol) Assessed In Vitro

**DOI:** 10.3390/molecules24010137

**Published:** 2018-12-31

**Authors:** Johanna Weiss

**Affiliations:** Department of Clinical Pharmacology and Pharmacoepidemiology, Heidelberg University Hospital, Im Neuenheimer Feld 410, 69120 Heidelberg, Germany; johanna.weiss@med.uni-heidelberg.de; Tel.: +49-6221-5639402

**Keywords:** Samento, Banderol, *Otoba parvifolia*, *Uncaria tomentosa*, cat’s claw, *Borrelia*, herb–drug interaction

## Abstract

Samento (extract from *Uncaria tomentosa*) and Banderol (extract from *Otoba parvifolia*) have been demonstrated to have anti-inflammatory and antimicrobial properties, e.g., against different morphological forms of *Borrelia burgdorferi*. However, there is hardly any data on the pharmacological safety of these two herbal medicines. This in vitro study aimed at scrutinizing their possible characteristics as perpetrators in pharmacokinetic herbal–drug interactions. Inhibition of cytochrome P450 enzymes (CYPs) was quantified by commercial kits and inhibition of drug transporters by use of fluorescent probe substrates. Induction was quantified by real-time RT-PCR and activation of pregnane x receptor (PXR) and aryl hydrocarbon receptor (AhR) by reporter gene assays. Organic anion transporting polypeptide 1B1 (OATP1B1) (IC_50_ = 0.49 ± 0.28%) and OATP1B3 (IC_50_ = 0.65 ± 0.29%) were potently inhibited by Banderol, but only weakly by Samento. CYP3A4 was inhibited about 40% at a Samento concentration of 1%. Samento significantly induced mRNA expression of *CYP2J2*, *UGT1A3*, *UGT1A9*, *ABCB1*, and *SLCO1B1* and strongly activated PXR, but hardly AhR. In conclusion, the perpetrator profiles of Samento and Banderol for herb–drug interactions completely differ. Clinical studies are strongly recommended to clarify whether the effects observed in vitro are of clinical relevance.

## 1. Introduction

*Uncaria tomentosa* (cat’s claw), a Rubiacea found in Central and South America, represents an important medicinal plant especially in Peru, Brazil, and Central America [1,2,3,4]. The bioactivity is broad and there are numerous data demonstrating its anti-inflammatory, antioxidant, antimicrobial, antidiabetic, anticancer, and immunostimulant properties [4]. *Uncaria tomentosa* exists in two chemical types, which contain either tetracyclic oxindole alkaloids (TOA) or pentacyclic oxindole alkaloids (POA) [5]. In Western Europe, only the TOA-free form has been licensed for the treatment of rheumatoid arthritis (Krallendorn, Immodal Pharmaka, Volders, Austria), because data demonstrated that only POAs stimulate the immune system, whereas TOAs antagonize this effect [5].

*Otoba parvifolia*, a Myristicacea also growing in Central and South America, is traditionally used, e.g., by the Waorani Indians from Ecuador, to treat infections caused by mites and fungi [6]. Moreover, antiprotozoal activity for extracts of this plant has been shown [7].

Samento (a TOA-free extract from *Uncaria tomentosa*) and Banderol (an extract from *Otoba parvifolia*) have been demonstrated to be effective against different morphological forms (spirochetes, round bodies, and biofilm-like colonies) of *Borrelia burgdorferi* in vitro [8], one of the *Borrelia* species causing Lyme borreliosis [9]. Whereas doxycycline, one of the most commonly used antibiotics to treat Lyme borreliosis, was only efficient against the spirochetal form, both extracts and especially the combination of them were also effective against the round body forms and biofilms, which are considered to be the source of the persistent Lyme disease [10,11]. Although no clinical or prescription data have been published so far, Samento and Banderol represent herbal medicines often used by patients suffering from Lyme borreliosis. Moreover, its consumption is recommended, e.g., by the Borreliose and FSME Society Germany, as anti-inflammatory and antimicrobial concomitant therapy for borreliosis patients.

Concerning the safety of the clinical application of these two herbal medicines, there are only sparse data for *Uncaria tomentosa*, but no published data for *Otoba parvifolia*. The use of the former is reported to be non-toxic [5]. Irrespective of toxicity and adverse drug reactions, herb–drug interactions are also of special importance for the safe administration of herbal drugs [12]. Data indicate that extracts of *Uncaria tomentosa* inhibit the most important drug metabolizing enzyme cytochrome P450 3A4 (CYP3A4) [13], possibly explaining the published case report describing an interaction between this compound and the HIV protease inhibitors atazanavir, ritonavir, and saquinavir [14]. Beyond this, no data exist on possible influences of *Uncaria tomentosa* and *Otoba parvifolia* on drug metabolizing enzymes or drug transporters being important for drug–drug or herb–drug interactions. Thus, this in vitro study evaluated the effects of Samento and Banderol on the expression and activity of a broad set of drug transporting or metabolizing proteins.

## 2. Results and Discussion

### 2.1. Transporter Inhibition by Samento and Banderol

Neither Samento nor Banderol increased intracellular calcein fluorescence in L-MDR1 cells over-expressing human P-glycoprotein (P-gp), indicating lack of P-gp inhibition (data not shown). Since P388/dx cells are more suitable to detect weak P-gp inhibitors, we also tested both compounds in these cells and the corresponding parental cell line. However, also in this cell system, no significant increase in intracellular calcein fluorescence by Samento or Banderol was observed, verifying the lack of P-gp inhibition by these extracts (data not shown).

Breast cancer resistance protein (BCRP) was neither inhibited by Samento nor by Banderol as indicated by a lack of an increase of intracellular pheophorbide A fluorescence in MDCKII-BCRP cells over-expressing human BCRP (data not shown).

**Figure 1:**
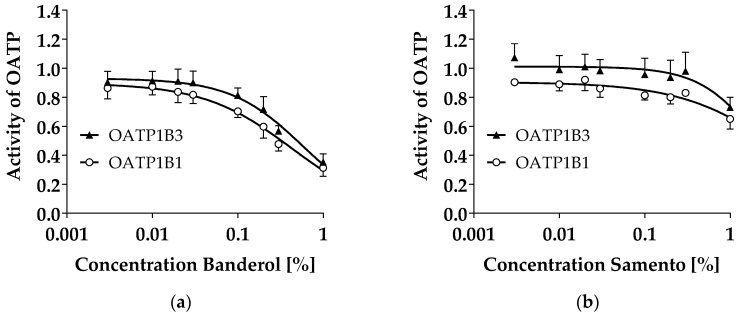
Concentration-dependent effect of Samento and Banderol on intracellular 8-cAMP fluorescence in HEK293 cells over-expressing organic anion transporting polypeptide (OATP)1B1 and OATP1B3, respectively, normalized to the mock-transfected control cell line. Data depict a biological triplicate ± S.E.M. with 30,000 cells measured by flow cytometry for each concentration in each experiment. (**a**) OATP inhibition by Banderol. (**b**) OATP inhibition by Samento.

Banderol concentration-dependently decreased intracellular 8-FcA fluorescence in organic anion transporting polypeptide (OATP)1B1 and OATP1B3 over-expressing HEK293 cells, indicating OATP1B1 and OATP1B3 inhibition with IC_50_ values of 0.49 ± 0.28% and 0.65 ± 0.29%, respectively (Figure 1A). Samento also inhibited both transporters, but with lower potency disabling a calculation of IC_50_ values (Figure 1B).

### 2.2. Inhibition of CYP1A2, CYP2B6, CYP2C19, and CYP3A4 by Samento and Banderol

**Figure 2:**
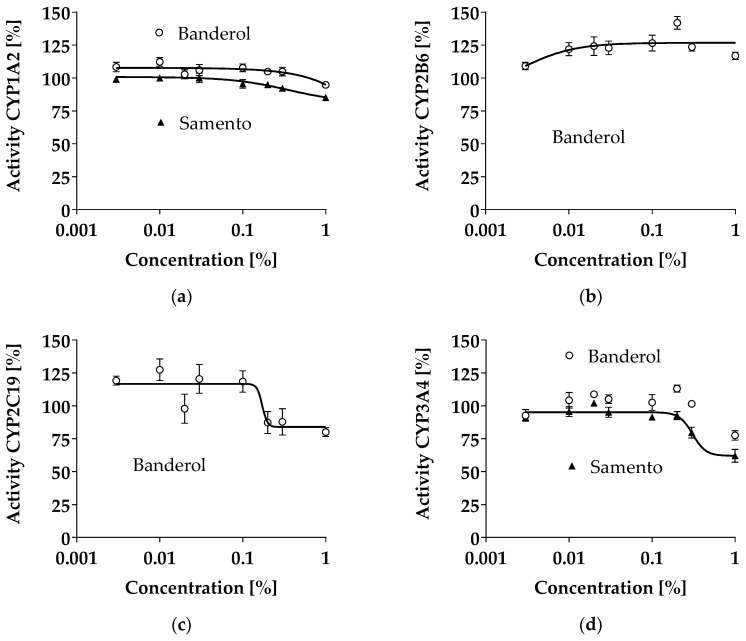
Concentration-dependent effect of Samento and Banderol on the activity of different cytochrome P450 enzymes (CYPs) quantified by P450-Glo™ Screening Systems. Each curve depicts the results of 3–4 experiments with each concentration tested in octuplet. Data are expressed as mean ± S.E.M. Samento did not change the activity of CYP2B6 and CYP2C19 and data are therefore not shown. (**a**) Inhibition of CYP1A2 by Samento and Banderol. (**b**) Inhibition of CYP2B6 Banderol. (**c**) Inhibition of CYP2C19 by Banderol. (**d**) Inhibition of CYP3A4 by Samento and Banderol.

CYP inhibition was tested with the respective P450-Glo™ Screening Systems. Up to the highest concentration tested, neither Banderol nor Samento profoundly inhibited CYP1A2, CYP2B6, CYP2C19, or CYP3A4 (Figure 2). The highest inhibition was demonstrated for CYP3A4 by Samento (around 40% at a concentration of 1%) and Banderol (around 20% at a concentration of 1%) and for CYP2C19 by Banderol (about 20% at a concentration of 1%).

### 2.3. Induction of Drug Transporters and Drug Metabolizing Enzymes by Samento and Banderol

**Figure 3:**
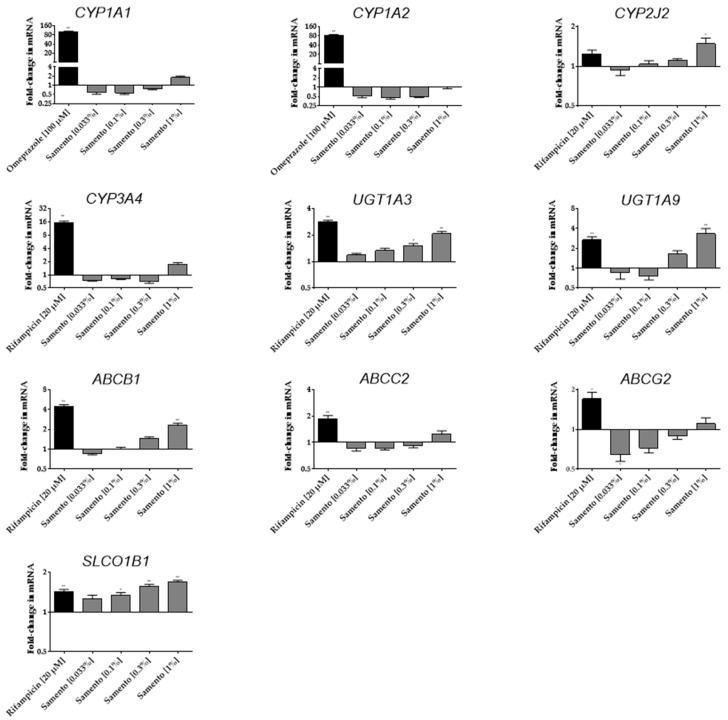
Influence of Samento on the mRNA expression of several drug metabolizing enzymes and drug transporters in LS180 cells. Cells were incubated for four days with different concentrations (0.033–1%) of Samento, or the positive controls rifampicin (20 µM) and omeprazole (100 µM), or medium alone (negative control). After harvesting, RNA extraction, and cDNA synthesis, mRNA was quantified by RT-PCR. Expression data were normalized to the housekeeping gene *GU* and to the negative control. Data are expressed as mean ± S.E.M. for *n* = 5 biological replicates. Data were analyzed using ANOVA with Dunnett’s post hoc test compared to the medium control. * *p* < 0.05, ** *p* < 0.01.

Induction of several drug metabolizing enzymes and drug transporters was tested by incubation of LS180 cells with Samento, Banderol, or the positive controls for four days. Figure 3 depicts the influence of Samento on the mRNA expression of several important drug metabolizing enzymes and drug transporters. The highest concentration of Samento tested (1%) significantly induced the mRNA expression of *CYP2J2*, *uridine diphosphate glucuronosyl transferase (UGT)1A3*, *UGT1A9*, *ABCB1*, and *SLCO1B1*. The lowest concentration of Samento tested (0.033%) repressed mRNA expression of *ABCG2* by 36%. No significant effects were observed on *CYP1A1*, *CYP1A2*, *CYP3A4*, and *multidrug resistance-associated protein 2* (*MRP2, ABCC2)* expression.

In contrast, Banderol had no significant effect on the mRNA expression of any of the genes investigated (data not shown).

### 2.4. Activation of PXR and AhR

**Figure 4:**
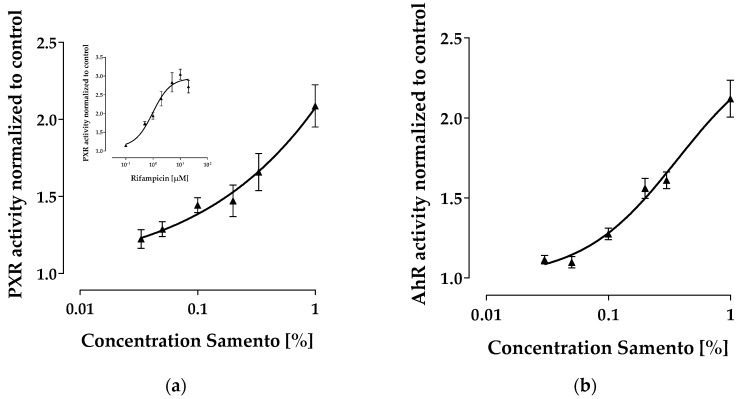
Results of the reporter gene assays. Each curve depicts the results of three experiments with each concentration tested in triplicate. (**a**) Concentration-dependent effect of Samento and the positive control rifampicin (insert) on pregnane x receptor (PXR) activity. (**b**) Concentration-dependent effect of Samento and the positive control omeprazole (insert) on aryl hydrocarbon receptor (AhR) activity.

Samento significantly induced mRNA expression of genes that are regulated by the pregnane x receptor (PXR) (e.g., *ABCB1*, *UGT1A3*, *UGT1A9*, *SLCO1B1*). We therefore investigated by means of reporter gene assay, whether this extract can activate PXR. Indeed, Samento concentration-dependently increased PXR activity, whereas at the highest concentration tested (1%) the maximum effect was obviously not reached (Figure 4A). To demonstrate possible influences on aryl hydrocarbon receptor (AhR), another important transcription factor regulating e.g., CYP1A, we also tested whether Samento can activate AhR and found only a minor effect compared to the positive control omeprazole (Figure 4B).

### 2.5. Influence of Samento on the Protein Expression of P-gp and CYP2J2

**Figure 5:**
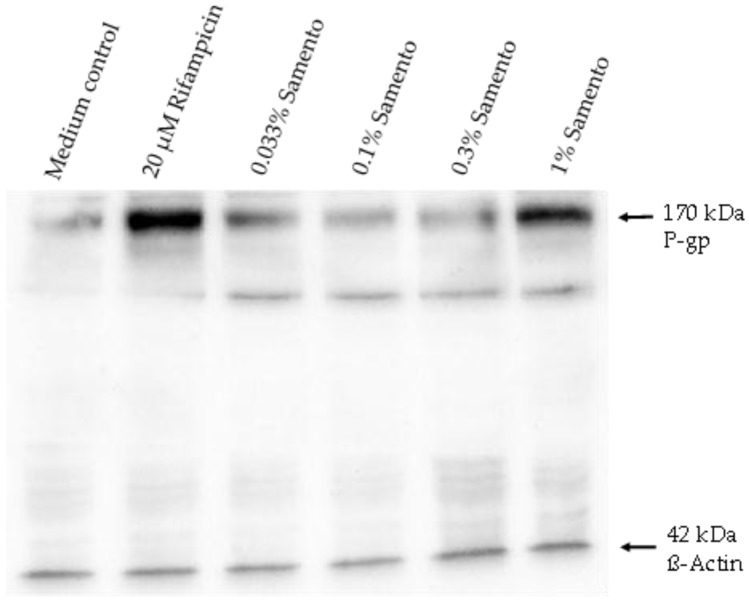
Effects of Samento (0.033–1%) and the positive control rifampicin (20 µM) on the protein expression of P-glycoprotein (P-gp) after four days of exposure. β-actin was used as a loading control. Exemplarily, 1 blot of a series of 5 is depicted.

Induction of P-gp by 1% Samento was verified at the protein level (Figure 5). In contrast, the small induction of *CYP2J2* at the mRNA level (1.5-fold) was not observed at the protein level (data not shown).

### 2.6. Influence of Samento on CYP2J2 mRNA Decay

**Figure 6:**
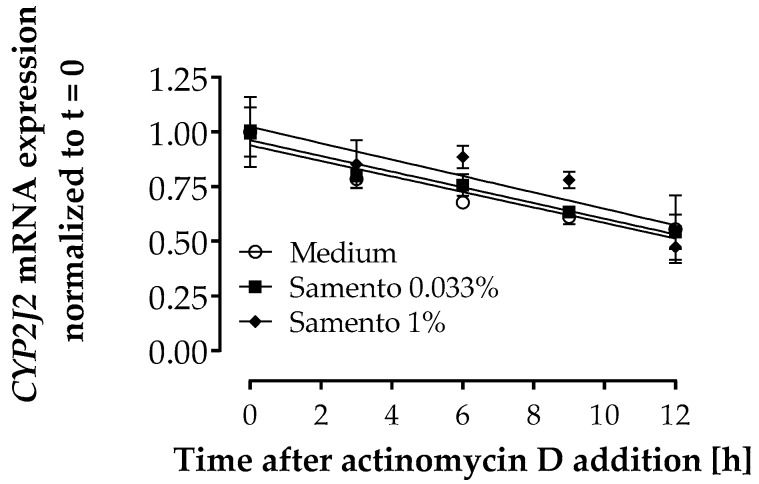
Effect of Samento on *CYP2J2* mRNA decay in LS180 cells. Cells were pre-treated with Samento (0.033 or 1%) or medium only for 12 h. Transcription was then inhibited by addition of 5 µg/mL actinomycin D and cells were harvested and RNA extracted after 3, 6, 9, or 12 h of treatment. Expression of *CYP2J2* mRNA was quantified by RT-PCR and results normalized to *β2mg* and to t = 0. Data are expressed as mean ± S.E.M. for *n* = 3 biological replicates.

Samento did not influence mRNA half-life of *CYP2J2* (Figure 6) indicating that the increase in mRNA expression observed with 1% Samento (Figure 3) can be attributed to transcriptional regulation and not to posttranscriptional mechanisms.

### 2.7. Discussion

So far, Samento (a TOA-free extract from *Uncaria tomentosa*) and Banderol (an extract from *Otoba parvifolia*) often used alternately or in addition to antibiotics by chronic borreliosis patients have not been characterized for their potential to act as perpetrators in herb–drug interactions. However, for safe application, it is crucial to know whether the pharmacokinetics of concomitantly used drugs can be altered by these herbal extracts. This study, therefore, scrutinized in vitro whether Samento and Banderol can inhibit the activity or induce the expression of important drug metabolizing enzymes and drug transporters.

This data for the first time demonstrates that both extracts do not inhibit the drug efflux transporters P-gp and BCRP but inhibit the liver uptake transporters OATP1B1 and OATP1B3. Inhibition of the OATPs was very weak by Samento but considerable by Banderol (Figure 1). Whether this inhibition is clinically relevant cannot be estimated, because it is completely unknown which ingredients at what concentrations reach the systemic circulation after ingestion of these herbal drugs.

Inhibition of the CYPs tested was mostly weak or absent up to the maximum concentration tested (Figure 2). The most profound effect was observed for CYP3A4 inhibition by Samento, reaching about 40% at a concentration of 1%, roughly matching the data obtained earlier with another extract of *Uncaria tomentosa* (CYP3A4 inhibition IC_50_ 0.8% [13]). Again, the clinical relevance is unclear due to the lack of pharmacokinetic data of Samento. However, at the highest recommended dose, the concentration of Samento in the gut is about 0.7%, potentially leading to intestinal CYP3A4 inhibition. This might explain the observed interaction of *Uncaria tomentosa* with HIV protease inhibitors [14] and might also lead to further interactions with concomitantly used drugs in borreliosis therapy, like clarithromycin, a CYP3A4 substrate [15]. Since CYP3A4 is the most important drug-metabolizing enzyme and its inhibition might lead to increased systemic exposure to every second licensed drug, this CYP3A4 inhibitory potential of Samento should be further evaluated in a clinical trial.

Not only inhibition, but also induction of pharmacokinetically relevant enzymes and transporters can lead to drug–drug or herb–drug interactions [16]. PXR activating drugs (e.g., rifampicin) or herbal preparations (e.g., St. John’s Wort) consequently induce drug metabolizing enzymes, and drug transporters can deteriorate drug efficacy by reducing the bioavailability or increasing the clearance of concomitantly used drugs, being substrates of such enzymes and/or transporters [16,17]. Whereas Banderol had no effect on any gene expression investigated, Samento turned out to clearly activate PXR in a similar range as the positive control rifampicin. In contrast, AhR was not profoundly affected (Figure 4). This is in good agreement with the observed mRNA inductions of PXR-driven genes (e.g., *ABCB1*) (Figure 3). The decrease of the PXR- and AhR-driven *ABCG2* expression at lower concentrations of Samento, however, is unclear. Possibly, induction provoked by one ingredient is superimposed by a repressive effect of another ingredient, leading to a net repressive effect at lower concentrations of Samento and a zero net effect at higher concentrations.

Interestingly, *CYP2J2* mRNA was also transcriptionally induced about 1.5-fold at the highest concentration of Samento tested. This induction was not observed at the protein level. However, this might be attributed to the much lower sensitivity of western blot semi-quantification compared to quantitative RT-PCR. So far, only few data exist on the regulation of *CYP2J2* expression. Our data indicate that a regulation via PXR seems unlikely, because the prototypical PXR activator rifampicin had no significant effect on *CYP2J2* expression (Figure 3). Moreover, there are no characteristic response elements for PXR in the *CYP2J2* upstream region [18], making it unlikely that PXR plays a major role in regulating *CYP2J2* expression. Other studies suggest that activator protein-1 (AP-1) and an AP-1-like element play a role in inducing *CYP2J2* expression in human liver-derived cells [18], which might also be the case in our cell system. *CYP2J2* is mainly expressed in the heart and plays an important role in arachidonic acid metabolism and thus in cardiovascular physiology [18]. In contrast, its involvement in induction-mediated drug–drug interactions is unknown so far, although several drug substrates have been described (e.g., ebastine, terfenadine, apixaban [18]), making this cytochrome another potential modulator of bioavailability.

Besides such drug-metabolizing enzymes, induction of *ABCB1* by Samento could also lead to reduced bioavailability of some of the frequently used antibiotics in borreliosis therapy, which are P-gp substrates: tetracycline [19], minocycline [20], azithromycin [21], and clarithromycin [21].

Unfortunately, it is unknown whether active compounds of Samento reach relevant systemic concentrations leading to hepatic induction of the genes investigated, but intestinal concentrations should be high enough to induce genes such as *ABCB1*, *CYP2J2*, *UGT1A3*, and *UGT1A9*.

## 3. Materials and Methods

### 3.1. Materials

Phosphate buffered saline (PBS), cell culture media, supplements, fetal calf serum (FCS), omeprazole, the cytotoxicity detection kit (LDH), and the GenElute™ Mammalian Total RNA Miniprep Kit were purchased from Sigma-Aldrich (Taufkirchen, Germany). Rifampicin, dimethyl sulfoxide (DMSO), Tris-hydroxymethyl-aminomethane (TRIS), dithiothreitol (DTT), Tween^®^20, and crystal violet were obtained from AppliChem (Darmstadt, Germany). The antibody against human P-glycoprotein (P-gp) clone C219 was from Calbiochem (Darmstadt, Germany), the secondary donkey anti-goat antibody was from Santa Cruz (Heidelberg, Germany), the secondary anti-mouse antibody was from GE Healthcare (Freiburg, Germany), and Rotiphorese^®^ gel 30 was obtained from Carl Roth GmbH (Karlsruhe, Germany). *CYP2J2* antibody (clone ab82361) was purchased from Abcam (Cambridge, UK). Actinomycin D was from Santa Cruz (Heidelberg, Germany). Pheophorbide A was from Frontier Scientific Europe (Carnforth, UK), calcein acetoxymethylester was from Invitrogen (Karlsruhe, Germany), and 8-fluorescein-cAMP (8-FcA) was from BIOLOG Life Science Institute (Bremen, Germany). The RevertAid™ H Minus First Strand cDNA Synthesis Kit, the Absolute QPCR SYBR Green Mix, and the Pierce ECL Western Blotting substrate were from Thermo Fisher Scientific (Waltham, MA, USA). Primers were synthesized by Eurofins MWG Operon (Ebersberg, Germany). The P450-Glo™ CYP1A2 Screening System, the P450-Glo™ CYP2B6 Screening System, the P450-Glo CYP2C19 Screening System, the P450-Glo™ CYP3A4 Screening System with Luciferin-IPA, Dual-Glo™, and the Steady-Glo™ Luciferase Assay System were obtained from Promega Corporation (Madison, USA). Samento and Banderol were obtained from Nutramedix (Jupiter, FL, USA).

### 3.2. Stock Solutions and Rationale for the Used Dilutions

Samento represents an extract from *Uncaria tomentosa* bark containing 22% ethanol. Banderol is an extract from *Otoba parvifolia* bark containing 20–24% ethanol. The maximum concentration used for both extracts was set to a dilution of 1:100 due to several reasons: (1) this dilution contains 0.2% ethanol, which does not influence the assays conducted; (2) dilutions of 1:400 showed best activity against the round-body forms of *Borrelia burgdorferi* in vitro, whereas higher concentrations were only effective against the spirochete form [8]; (3) the manufacturer of Samento and Banderol recommends to take a maximum of 30 drops (=1.5 mL) in about 100 mL water, which represents a dilution of 1:150 (=0.7%). Thus, it seemed unreasonable to test higher concentrations than a dilution of 1:100 (=1%).

### 3.3. Cytotoxicity Assays

Toxic effects on cells can negatively influence transporter inhibition assays. Thus, prior to transporter inhibition assays, Samento and Banderol were investigated for their cell-toxic effects using the cytotoxicity detection kit (LDH) based on the release of lactate dehydrogenase according to the manufacturer’s instruction. Samento and Banderol were revealed to be non-toxic up to the maximum tested concentration (1%) in all cell lines used.

### 3.4. P-gp Inhibition Assay

Possible P-gp inhibition by Samento and Banderol was assessed in a calcein assay using calcein acetoxymethylester as a fluorogenic substrate as validated and described previously [22,23]. As cell models, we used L-MDR1 cells over-expressing human P-gp and its wild-type counterpart LLC-PK1 and P388/dx cells over-expressing murine P-gp and the corresponding parental cell line P388. Cell lines were cultured and treated as published previously as were the results for the positive controls verapamil and quinidine [22,23], which are not depicted in this publication, because the potency of a small molecule (given in molar) cannot be compared to that of an extract (given as a dilution).

Each concentration of Samento and Banderol (0.001–1%) was tested in octuplet and each experiment was performed in triplicate.

### 3.5. BCRP Inhibition Assay 

BCRP inhibition was tested by flow cytometry using pheophorbide A as a specific BCRP substrate in MDCKII-BCRP cells compared to the parental cell line MDCKII as described previously [24]. Results for the positive controls were also published previously [24]. Concentrations ranging from a dilution of 0.0033 to 1% were tested and the experiment was performed in triplicate.

### 3.6. OATP Inhibition Assay

Inhibition of OATP1B1 and OATP1B3 was quantified by flow cytometry quantifying the uptake of 8-FcA into HEK293 cells over-expressing the respective transporter in comparison to HEK293 cells transfected with the empty control vector as published previously [25]. Concentrations ranging from a dilution of 0.0033 to 1% were tested and the experiment was performed in triplicate. Results for the positive controls rifampicin and cyclosporine A were published previously [25].

### 3.7. Inhibition of CYP1A2, CYP2B6, CYP2C19, and CYP3A4

Inhibition of CYPs was assessed with the P450-Glo™ Screening Systems according to the manufacturer’s instructions. The kits contain a luminogenic substrate (luciferin-ME for CYP1A2, luciferin-2B6 for CYP2B6, luciferin-h EGE for CYP2C19, or luciferin-IPA for CYP3A4), which is converted by the respective enzyme into luciferin. When incubated with the luciferin detection reagent, the luciferase generates light. Banderol and Samento were tested for their capacity to inhibit the production of the luminescent signal. Positive controls used were ketoconazole for CYP3A4, omeprazole for CYP2C19, miconazole for CYP2B6, and β-naphthoflavone for CYP1A2. Results are not depicted, because the potency (given in molar) of a small molecule cannot be compared to that of an extract (given as a dilution).

Eight concentrations in triplicates ranging from 0.0033 to 1% were tested and each experiment was conducted 3–5 times.

### 3.8. Growth Inhibition Assay

Prior to induction experiments, antiproliferative effects of Samento and Banderol were investigated in the cell lines used (LS180 cells) to exclude an influence on cell growth. Proliferation was quantified by crystal violet staining and the assays were conducted as described previously [26]. Each concentration (dilution 0.001–1%) was tested in octuplet and each experiment was performed in quadruplicate. Both extracts had no effects on cell proliferation up to the maximum concentration tested (1%). Therefore, Samento and Banderol were tested up to a dilution of 1% in the induction assay.

### 3.9. Induction Assay

For induction experiments, the human colon adenocarcinoma cell line LS180 (available at ATCC, Manassas, VA, USA) was used. This cell line is a suitable surrogate for the intestine being a major site of drug interactions and it is ideal for investigating AhR and PXR mediated induction [27]. Cells were cultured under standard cell culture conditions as published previously [27].

Three days before starting the assay, LS180 cells were seeded in culturing flasks. Medium was then changed to medium containing either Samento or Banderol (dilution 0.033%, 0.1%, 0.33%, 1%) and cells were incubated for four further days. Rifampicin (20 µM) served as a positive control for PXR-driven genes, and the AhR ligand omeprazole (100 µM) for CYP1A1 and CYP1A2 induction, and compound-free medium as a negative control. All media were adjusted to 0.03% DMSO. After harvesting, RNA was extracted immediately. Each experiment was conducted in quintuplicate.

### 3.10. Quantification of mRNA Expression by Real-Time RT-PCR

RNA was isolated using the GeneElute Mammalian Total RNA Miniprep Kit and cDNA was synthesized with the RevertAid™ H Minus First Strand cDNA Synthesis Kit according to the manufacturers’ instructions. Gene expression was quantified by real-time RT-PCR as described previously [28] for the following genes: *CYP1A1*, *CYP1A2*, *CYP2J2*, *CYP3A4*, *UGT1A3*, *UGT1A9*, *ABCB1*, *ABCC2*, *ABCG2*. For *UGT1A3* a Quantitect Kit (Qiagen, Hilden, Germany) was used according to the manufacturer’s instructions and primers for *CYP2J2* were published by another group [29]. All other primer sequences and PCR conditions were published previously [27].

The most stable housekeeping gene for normalization was identified using geNorm (version 3.4, Center for Medical Genetics, Ghent, Belgium), which determines most stable reference genes from a set of tested genes in a given cDNA sample panel [30]. Among a panel of 7 housekeeping genes tested, *glucuronidase β* (*GU*) was the most stable under the treatment with Samento, and *glucose-6-phosphate dehydrogenase* (*G6PDH*) under the treatment with Banderol and were thus used for normalization. Primer sequences and conditions were published previously [31]. Data were evaluated as described previously [28]. All samples were amplified in duplicate and the mean of the technical duplicate was used for further calculation.

### 3.11. Western Blot Analysis of P-gp and CYP2J2 Expression

To verify gene induction, one of the PXR-driven genes, P-gp, was also analyzed at the protein level. Moreover, since the regulation of *CYP2J2* is largely unknown, its up-regulation by Samento was also investigated at the protein level. Protein expression was quantified by SDS-PAGE and western blotting according to standard protocols as published previously [25]. Protein detection was carried out with a murine monoclonal antibody against human P-gp (clone C219, diluted 1:100 in TRIS-buffered saline containing 0.1% Tween^®^20), or human *CYP2J2* (clone ab82361, diluted 1:50), or β-actin (Clone AC-74; diluted 1:40,000). After extensive washing, blots were incubated with horseradish peroxidase-linked secondary antibodies and bands were visualized by chemiluminescence using the Pierce ECL Western Blotting Substrate and semi-quantified by FluorChem Q SA AlphaView Version 3.2.2, Cell Biosciences (Santa Clara, USA). Expression was normalized to the loading control β-actin and the untreated medium control. Each blot was conducted in quintuplicate.

### 3.12. PXR Reporter Gene Assay

To investigate whether inductions of typical PXR-driven genes, like *ABCB1*, by Samento truly originate from activation of PXR, a PXR reporter gene assay was conducted in LS180 cells using the Dual-Glo™ Luciferase Assay System according to the manufacturer’s instructions as published previously [25]. Increases of PXR activity after 24 h incubation with Samento or the control compound rifampicin were normalized to the transfection efficiency control (renilla luminescence) and to the PXR activity of non-treated controls set to 1 (=100%). The assay was conducted in triplicate.

### 3.13. AhR Reporter Gene Assay

To investigate whether Samento can also activate AhR, a reporter gene assay for AhR in AZ-AhR cells stably transfected with a construct containing several AhR binding sites upstream of a luciferase reporter gene was conducted using the Steady-Glo™ Luciferase Assay System according to the manufacturer’s instructions as described previously [27]. Compound-induced increases of AhR activity were normalized to the activity of non-treated controls (set to 1 = 100%). The assay was conducted in triplicate and omeprazole was used as a positive control.

### 3.14. Evaluation of the Influence of Samento on CYP2J2 mRNA Decay

To investigate whether the increase of *CYP2J2* mRNA under treatment with Samento can be attributed to posttranscriptional regulation, we determined whether Samento influences the mRNA decay of this gene. LS180 cells were treated with Samento (0.033 or 1%) or medium only for 12 h. Afterwards, transcription was inhibited by addition of 5 µg/mL actinomycin D and cells were harvested and RNA extracted after 3, 6, 9, or 12 h of treatment.

mRNA was quantified by qRT-PCR as described above. Since *β2-microglobulin* (*β2mg*) revealed to be most stable among a set of housekeeping genes tested under treatment with actinomycin D, it was used for normalization.

### 3.15. Statistical Analysis

Data were analyzed using GraphPad Prism Version 7.02 and InStat Version 3.06 (GraphPad Software, San Diego, USA). IC_50_ values were calculated using the four-parameter fit (sigmoidal concentration-response curves with variable slope). Differences between mRNA expression following incubation with the investigated compounds and the respective vehicle controls were tested using ANOVA with Dunnett’s post hoc test. A *p*-value < 0.05 was considered significant.

## 4. Conclusions

In conclusion, Samento and Banderol exhibit a completely different in vitro profile as perpetrators in herb–drug interactions. Whereas Banderol crystallized as a potent inhibitor of the uptake transporters OATP1B1 and OATP1B3, Samento’s inhibitory potential on CYP3A4 and its property as a PXR agonist and thus as a gene inducer warrant caution when using these herbal extracts concomitantly with other drugs. Clinical studies are strongly recommended to clarify whether the effects observed in vitro translate into the clinical setting.
